# Chinese Women’s Concept of Childbirth Based on the Social Media Topic “What Does Childbirth Mean to a Woman”: Content and Thematic Analysis

**DOI:** 10.2196/50512

**Published:** 2024-01-05

**Authors:** Ting Yang, Yihan Wu, Nuo Han, Tianli Liu

**Affiliations:** 1 Institute of Population Research Peking University Beijing China; 2 Graduate School of Education Peking University Beijing China; 3 Chinese Academy of Sciences Key Laboratory of Behavioral Science Institute of Psychology Chinese Academy of Sciences Beijing China; 4 Department of Psychology University of Chinese Academy of Sciences Beijing China; 5 School of Data Science City University of Hong Kong Hong Kong SAR China

**Keywords:** childbirth willingness, social media, risk perception, childbirth cost, childbirth benefit

## Abstract

**Background:**

In recent years, women’s fertility desire has attracted increasing attention in China.

**Objective:**

This study aims to detect attitudes toward giving birth among young female users on Douban, a very popular Chinese social media platform.

**Methods:**

A total of 2634 valid posts from 2489 users discussing the topic “What does childbirth mean to a woman” on Douban were crawled and retained for analysis. We utilized content and thematic analysis methods to capture users’ concepts of childbirth.

**Results:**

The findings reveal that a significant majority of users conveyed generally neutral (1060/2634, 40.24%) or negative (1051/2634, 39.90%) attitudes toward childbirth, while only about one-fifth of users expressed positive (523/2634, 19.86%) sentiments. Notably, posts with negative attitudes garnered more replies and likes, and the proportion of posts expressing negativity exhibited fluctuations over time. Health risk (339/2634, 12.87%) emerged as the most frequently cited aspect of childbirth cost, with subjective happiness and the fulfillment of mental needs identified as primary benefits. Surprisingly, only a minimal number of posts (10/2634, 0.38%) touched upon the traditional objective benefits of raising children for old-age care. Thematic analysis results suggest that discussions about fertility on social media platforms might contribute to an exaggerated perception of health risks among women. Additionally, a lack of knowledge about childbirth was observed, partially attributable to longstanding neglect and avoidance of communication on these matters, likely influenced by traditional cultural biases. Moreover, there is a prevailing assumption that women should naturally sacrifice themselves for childbirth and childcare, influenced by the idealization of the female figure. Consequently, women may harbor hesitations about having a baby, fearing the potential loss of their own identity in the process.

**Conclusions:**

The results indicate a shift in the perception of childbirth among modern Chinese women over time, influenced by their increasing social status and the pursuit of self-realization. Implementing strategies such as public education on the health risks associated with pregnancy and delivery, safeguarding women’s rights, and creating a supportive environment for mothers may enhance women’s willingness to undergo childbirth.

**International Registered Report Identifier (IRRID):**

RR2-10.2196/preprints.50468

## Introduction

The academic community in demographics has centered its attention on fertility issues, and China is currently grappling with a significant challenge in population development due to its low fertility rate. In August 2020, the National Health Commission of the People’s Republic of China issued a document highlighting that the low fertility rate has emerged as a major risk impacting the balanced development of the country’s population. To mitigate the consequences of population aging and the decline in demographic dividends resulting from the low fertility rate, China introduced the “Universal Two-Child Policy” in January 2016, followed by the “Three-Child Policy” in May 2021. The implementation of these policies has elevated individual childbirth willingness as a crucial factor influencing the childbirth rate [1]. The determinants of childbirth are multifaceted and intricate; past research indicates that cultural norms and values play a substantial role in shaping both the concept and behavior associated with childbirth [2,3]. Research on the willingness to have a second child among Chinese women has indicated that cultural concepts play a pivotal role in influencing the decision to pursue a second child [4]. In the context of a highly “mediatized” modern societal culture, where the media not only exerts influence but also, to some extent, shapes cultural attitudes [5], it appears that women’s fertility desires are more significantly impacted by media exposure compared with men [6].

In contemporary society, the internet has emerged as one of the foremost cultural media. As of June 2022, the number of internet users in China had surpassed 1.051 billion, with an adoption rate of 74.4% [7]. Serving as a crucial platform for individuals to articulate their perspectives, the internet fosters cultural diversity and the expression of values. This, in turn, influences personal attitudes toward marriage, inspiring individuals to seek independence, personal happiness, and a heightened awareness of emotional connection and respect for individuality [8]. The evolution of the internet is concurrently driving a shift in the concept of gender roles among rural residents, transitioning from traditional to modern perspectives. This influence is evident in both men and women, with a notably more pronounced effect on women compared with men [9]. Social media, as a novel form of online communication, provides users with an extensive platform for active participation. It embodies features such as engagement, openness, real-time communication, community-building, and connectivity [10]. Social interaction serves as a crucial mechanism by which media can shape fertility behaviors and concepts [11]. Consequently, the utilization of social media can influence people’s perceptions of fertility and their intentions regarding it. Examining discussions about fertility on social media becomes valuable in gaining insights into the collective understanding of fertility among the populace.

In recent years, a growing number of women have been actively sharing comments on childbirth-related topics, including discussions about the “2-child” and “3-child” policies, expressing their personal insights and thoughts on the matter. A notable distinction is that the majority of discussions on foreign social media platforms exhibit a predominantly positive attitude toward childbirth [12,13]. Nevertheless, the discourse on fertility in China’s social media landscape is characterized by a prevalence of antifertility sentiments [14]. On the one hand, the textual information available on the internet partially mirrors the childbirth concepts held by contemporary women. On the other hand, public opinion expressed on social media platforms may exert an influence on women’s perceptions of childbirth, potentially diminishing their willingness to pursue childbirth [15,16]. In this study, content analysis was used to delineate the predominant attitudes evident in posts within a social media topic focused on childbirth. Additionally, the analysis aimed to identify the costs and benefits associated with childbirth that garnered attention and concern among the participants. Furthermore, thematic analysis was utilized to reveal the underlying childbirth themes embedded in the posts under this particular topic.

## Methods

### Sample

#### Selection of Research Platform

This paper selected Douban (Beijing Douwang Technology Co. Ltd.) as the source platform for data collection based on the considerations outlined in Textbox 1.

**Considerations for data collection.**

**Figure 1:**
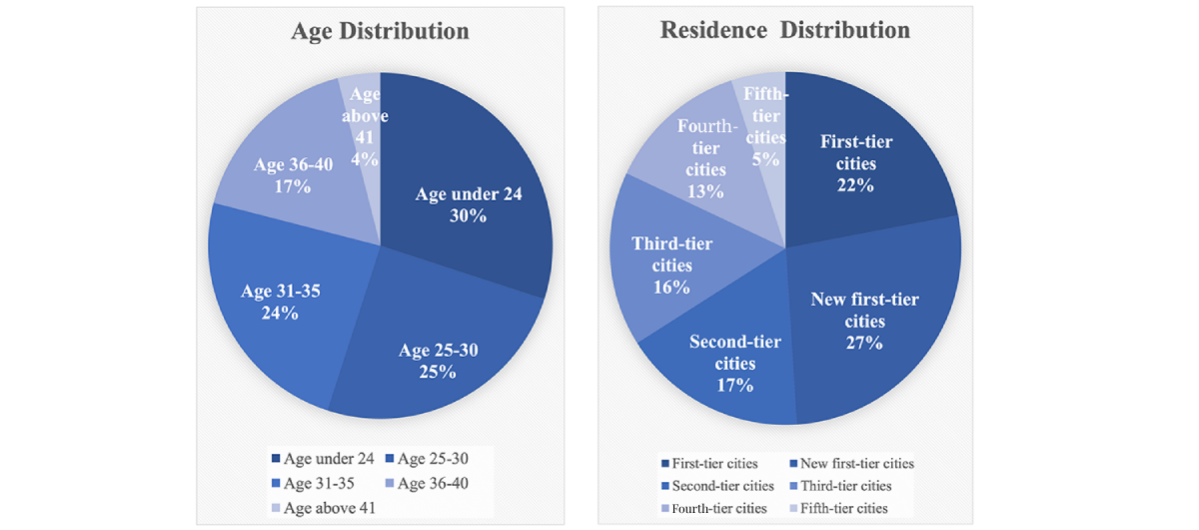
User groups in May 2022 according to the “Qianfan” query platform.

1. To identify suitable social media data for the study, the research team conducted a screening of childbirth topics across various popular platforms in China, such as Weibo (Weibo Corporation), WeChat (Tencent Holdings Limited), TikTok (ByteDance), Bilibili (Bilibili Inc.), and Douban. It was observed that the feature settings of Douban and Weibo rendered them more conducive to text analysis. However, on Weibo, no topics closely aligned with our research objective were found, and the word count and content quality of Weibo posts were found to be inferior to those on Douban.

2. Douban stands out as a popular social media platform, particularly among young women of childbearing age. As per data from the “Qianfan” query platform, which serves as a digital economy market information and data terminal, Douban boasts a monthly active user base of 10.51 million. Notably, within this user demographic, women constitute a significant majority at 63.3%. In comparison to other social media platforms such as Weibo and WeChat, Douban exhibits a higher proportion of female users. The bulk of Douban’s user demographic falls within the childbearing age range, with 30.07% aged under 24 years, 24.83% aged 24-30 years, and 24.25% aged 30-35 years. Additionally, 49.14% of users originate from first-tier or new first-tier cities. This demographic profile underscores that a significant proportion of Douban users are urban women of childbearing age. Consequently, their discussions on childbirth have the potential to reflect the attitudes and concerns toward childbirth among Chinese women to a considerable extent. It is noteworthy that many studies focusing on Chinese women have selected Douban as their research platform (Figure 1, [17-20]).

#### Selection of Research Topic

Among the numerous childbirth-related topics on Douban, this paper selected the one most pertinent to women’s concept of childbirth and with the highest number of posts. Specifically, the chosen topic is “What does childbirth mean to a woman.” Initiated in June 2020, this discussion has garnered significant engagement, accumulating 3655 posts as of August 5, 2022. Notably, the most popular post on this topic received 5596 likes. The substantial quantity and the evident quality of the posts led us to conclude that they met our inclusion standards.

#### Selection of Posts Under the Research Topic

This study focused on analyzing the discussions within the topic “What does childbirth mean to a woman,” and data were collected by crawling posts from June 10, 2020, to July 16, 2022. A total of 3403 raw posts from 2838 users were gathered, encompassing information such as post time, content, and likes. Additionally, permanent residence details were obtained for 2128 users. Recognizing the contextual nature of replies below the posts and the challenges associated with their identification and categorization, we opted to solely crawl the original posts. Replies beneath the posts were not included in the data collection process, acknowledging the potential complexity in comprehending their content without the necessary context from the original posts.

The crawled posts underwent a standardized screening and cleaning process, which unfolded in 2 steps. Initially, 2 categories of invalid data were systematically removed. The first category encompassed elements such as emojis, symbols, pictures, and blanks, which were deemed unsuitable for text analysis. The second category included irrelevant posts or those with an ambiguous attitude toward childbirth, such as advertisements, personal life records, ambiguous sentences, questions, and posts from men, among others. Following these criteria, a total of 460 posts were eliminated in the first step of the cleaning process. In the second step, for users who posted more than once, we analyzed their attitude toward childbirth. If all posts from the same user exhibited a consistent attitude, the post with the highest number of likes was retained. In cases where posts had the same number of likes, the one with the greater word count was preserved, and any remaining posts were eliminated. This approach aimed to distill the most representative content from users with multiple posts on the same topic. If a user’s attitude toward childbirth changed, the post with the highest number of likes under each different attitude was retained. For instance, if a user’s attitude shifted from positive to neutral and then to negative, 3 posts would be retained. Adhering to this criterion, a total of 309 posts were sequentially eliminated. Importantly, to maintain the focus on women’s concepts of childbirth, all posts where the posters identified as male were removed. While the data did not explicitly specify the gender of the posters, given the topic’s nature, the predominant female user base on Douban, and the thorough data cleaning process, it is argued that most of the retained posts after elimination originated from women. In the end, 2634 valid posts from 2489 users were retained for analysis.

### Ethics Approval

To safeguard user privacy, this study used the practice of using the first letter of each word in the username to replace the full name. For English usernames, the first 3 letters were utilized. Additionally, the post time was appended after each post. The study adheres to ethics code H15009 and obtained approval from the Institutional Review Board at the Institute of Psychology at the Chinese Academy of Sciences.

### Content Analysis Procedure

Content analysis aims to describe the prevailing attitudes of people toward childbirth and their concerns about the costs and benefits associated with childbirth. We referenced the methods from MacPherson et al [21] and Liu et al [22]. Two investigators (TY and YHW), with an in-depth understanding of Douban’s cultural environment, conducted the content analysis. Initially, the 2 researchers read 10% (340/3403) of the posts and then engaged in discussions to generate coding criteria for attitudes toward childbirth, the costs of childbirth, and the benefits of childbirth (Tables 1, 2 and 3). Subsequently, they randomly selected 50 posts to verify the saturation of the coding criteria. Following that, both researchers independently coded all the data based on the coding criteria. During this process, they conducted regular cross-checks to ensure the consistency of their coding results, and any disagreements were adjudicated by the third researcher (NH). After that, quantitative descriptive results concerning childbirth attitudes, childbirth costs, and childbirth benefits were obtained.

**Table 1:** Coding criterion for attitudes toward childbirth.

Category and definition			Example
**Negative**			
	Willingness not to give birth or regret to give birth	If I had known that childbirth would deprive the freedom to live an unconstraint life, to cook, to attend an exhibition, and to sleep late at weekends, to improve myself and play at night, I truly would not have a baby. [Poster and Time: CC, June 23, 2020, 16:21:08]	
	Negative effects of childbirth	It means my belly not going back to how it was before I was pregnant, anterior pelvic tilt, fat saddlebags, diastasis recti abdominis, sagging breasts and no freedom. [Poster and Time: LTGZ, October 12, 2020, 15:02:29]	
	Sharing of personal experiences or stories that makes researchers feel subjective negative attitudes toward childbirth	Today, a colleague returned to work after maternity leave. Her office cubicle was no longer available in the department. I asked if there was a need to register in new financial software for her, while the manager said he didn’t know. Then, the leader said to seal her employment separation certificate. What a sad story! [Poster and Time: PSH, April 27, 2021, 21:12:27]	
**Neutral**			
	A view that agrees with both giving and not giving birth	It means freedom and rights. As a woman, getting married or having children is only one of the choices. Whether to get married or not, whether to have children or not is our own choice. [Poster and Time: WZHYLDN, June 11, 2020, 15:07:05]	
	Positive or negative effects of childbirth with no indication of personal willingness	Having a baby means the loss of your own life. Isn’t taking care of children a part of your life? There is no accounting for tastes. Opportunities and costs are everywhere and need to be chosen. [Poster and Time: MISS, October 15, 2020, 08:43:17]	
	Sharing of personal experiences or stories does not make researchers feel subjective attitudes toward childbirth	I don’t have a child, but I have a friend who already has a baby. I see her state, there are gains and losses. What childbirth means to women may depend on their own state of mind/family attitudes. [Poster and Time: QHCD, November 28, 2020, 23:44:33]	
**Positive**			
	Willingness to give birth	Most likely, because my own family is incomplete, I personally want to get married and have children to start a new family. [Poster and Time: NKWKBKN, June 13, 2020, 04:31:45]	
	Positive effects of childbirth	For me, I am very happy, I have a sense of responsibility, one more person I care about, more maturity and stability. It is like a seed, growing with my care, and I have a sense of achievement and pride. [Poster and Time: XYCDXWB, June 11, 2020, 12:18:34]	
	Sharing of personal experiences or stories makes researchers feel subjective positive attitudes toward childbirth	I chatted with my friend last night, who was the same age as me. Her daughter is five years old, and I thought she would become complaining, grumpy and anxious like other mothers, but she doesn’t. She has a strong ability of introspection and awareness to change and adjust her state of mind. [Poster and Time: YYSH, June 17, 2020, 10:12:49]	

**Table 2:** Coding criterion for the costs of childbirth.

Category	Posts (N=2634), n (%)	Definition	Example
Health risks	339 (12.87)	Severe reactions during pregnancy, physical pain of childbirth, sequelae of childbirth, and postpartum depression	*After giving birth, I still feel pains in some part of my body. The arms, knees and heels feel cold even in summer, so that I have to wear socks to sleep every day. As the mother of two daughters, I hope that they will choose not to marry and be infertile in the future.* [Poster and Time: QQWDBB, June 18, 2020, 10:14:30]
Constraint on freedom	232 (8.81)	Constraint on freedom and the lack of self-personality caused by childbirth	*It means losing freedom and self within an uncertain period.* [Poster and Time: LSSJL, July 17, 2020, 22:08:34]
Energy investment	162 (6.15)	Energy investment during pregnancy and parenting, such as the inability to sleep due to breastfeeding	*After being a mother, 24 hours a day is not enough. I don’t want to sleep more, just want to fight for more time of my own.* [Poster and Time: CXMY, July 19, 2020, 08:50:38]
Influence on occupation	159 (6.04)	Workplace discrimination and the impact of childbirth on occupation	*Having a baby means your career will be forced to stagnate for 3 to 5 years. The tiredness and concern for children are really a major cost for women in the workforce.* [Poster and Time: BBQDJXK, April 18, 2021, 02:02:55]
Parenting responsibility	84 (3.19)	Responsibility for childbirth and education of children	*I don’t know why. But when I see the topic, the first thought coming into my mind is responsibility, being responsible for myself and for my baby. If I can’t do it well, I will not give birth. I don’t yearn for giving birth. And I will not regret if I don’t have any children.* [Poster and Time: CSG, June 11, 2020, 19:31:50]
Influence on appearance	84 (3.19)	Influence of appearance, figure, scar, etc	*After giving birth, it is easy to welcome the coming of a new baby but is difficult to face with the linea nigra, stretch marks on the thighs and flabby belly.* [Poster and Time: LKNT, May 1, 2021, 02:53:04]
Family relationship	69 (2.62)	Negative effects of family relationships caused by childbirth	*Many spouses will engage in emotional abuse. I have seen numerous examples around me, where after the wife gave birth, the husband became particularly distant and cold towards her.* [Poster and Time: DY, November 20, 2020, 01:38:22]
Financial investment	33 (1.25)	Financial investment due to childbirth	*My baby is 16 weeks old. I need to have the Down syndrome screening, Mediterranean anemia test, and an ultrasound. These tests will cost 953 yuan, which is really expensive......* [Poster and Time: PTR, December 30, 2021, 09:31:21]

**Table 3:** Coding criterion for the benefits of childbirth.

Category	Posts (N=2634), n (%)	Definition	Example
Parenting experience	196 (7.44)	Well-being, love, and happiness felt in the parenting process	*It is lucky for a woman to be able to deliver a baby, because only by giving birth can you know how happy it is to be a mother. A little baby who is as small as a meat ball can grow up after your care. And every progress he achieved will let you feel happy. I think companion is the best moment in the world.* [Poster and Time: HL, June 13, 2020, 17:37:32]
Self-growth	115 (4.37)	Growth of knowledge, the reconstruction of world outlook and values, and the maturity of self-character	*I think giving birth is an opportunity for a woman and a man to grow up. The deeper life goes, the greater the difference and the resistance to seeking common ground. Because of children, we have the desire to seek common ground, so the creativity to overcome resistance is stronger and the vitality is also stronger.* [Poster and Time: KGZDM, June 30, 2020, 17:09:09]
Continuation of life	38 (1.44)	The social value of propagation of the race and the significance of the personal continuation of life	*I chose to have children just because I think child is still a continuation of our blood in this world when my husband and I died, which can prove that we have been here. It is beautiful to think about it, isn’t it?* [Poster and Time: NL, June 16, 2020, 08:14:36]
Children’s company and psychological support	23 (0.87)	Children’s daily company, psychological sustenance, etc	*Parents accompany you in the first half of your life, and children do the same in the second half of your life. People will feel reassured when there is always a person accompanying you in your life.* [Poster and Time: TK, June 23, 2020, 12:18:10]
Complete life	22 (0.84)	The integrity of personal values and life experiences	*I think childbirth has completed a transformation from being a woman to a mother for me. It lets me realize the greatness and selflessness of my mother. It also makes my life as a woman more complete.* [Poster and Time: HX, June 15, 2020, 21:01:44]

### Thematic Analysis Procedure

Thematic analysis aims to explore more intricate childbirth concepts, providing an exploratory theoretical explanation for the results of content analysis. More specifically, the results of the thematic analysis can provide explanations and insights into why there is a widespread prevalence of negative childbirth attitudes, why some childbirth costs are of particular concern, and what kind of support individuals require from the government and society. While content analysis helps identify themes based on frequency, thematic analysis reveals potential themes within the data that can present a more nuanced perspective [23]. This study utilized the thematic analysis approach with the 5 phases outlined by Braun and Clarke [23]. In the first phase, the authors immersed themselves in the data through repeated readings and viewing, critically contemplating the meanings within the content of the posts. In the second phase, the research team identified initial codes. Moving into the third phase, the authors shifted from identifying codes to identifying themes, interconnecting the codes logically to form themes. The fourth phase involved reviewing each theme’s relation to the data overall and to the other themes to determine the boundaries of each theme. In the fifth phase, the research team defined, named, and elaborated on each theme and extracted illustrative examples of the final themes [24].

## Results

### Content Analysis

#### Basic Attitude

In the content analysis, we identified coding criteria related to attitudes toward childbirth, and Table 1 illustrates specific criteria and examples. The basic classification of the attitude toward childbirth showed that 1051/2634 (39.90%) posts had a negative attitude, 523/2634 (19.86%) posts had a positive attitude, and 1060/2634 (40.24%) posts had a neutral attitude. This indicates that the public attitude toward childbirth is generally neutral or negative, with fewer instances of a positive outlook. By extracting and classifying the top 10 most-liked posts, we found that there were 5 posts with a negative attitude (50%), 2 posts with a positive attitude (20%), and 3 posts with a neutral attitude (30%). The negative ratio was higher, but it was basically consistent with the whole distribution. From the word count, the posts with a positive attitude are the longest, but the number of likes is the lowest. By contrast, the posts with a negative attitude are the shortest, but the number of likes is the highest (Table 4).

**Figure 2:**
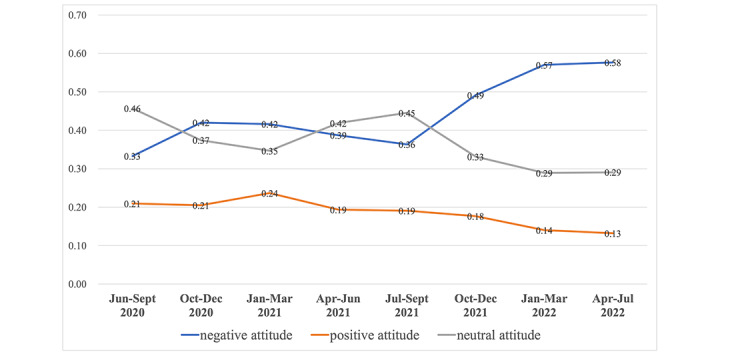
Changes in the proportion of three childbirth attitudes with time.

If the factor of time is taken into account and the changes in the proportion of childbirth attitudes in different periods are analyzed, it can be found that the proportion of posts with a negative attitude fluctuates to rise, the proportion of posts with a neutral attitude fluctuates to decline, and the proportion of posts with a positive attitude has small fluctuations and shows a downward trend (Figure 2).

**Table 4:** Distribution of childbirth attitude of posts.

Category	Posts (N=2634), n (%)	Top 10 most-liked posts, n (%)	The average number of words in 1 post	The median number of words in 1 post	The average number of likes in 1 post	The median number of likes in 1 post
Negative	1051 (39.90)	5 (50)	277.64	103	41.27	4
Neutral	1060 (40.24)	3 (30)	352.87	101	28.48	3
Positive	523 (19.86)	2 (20)	415.86	158	26.26	3

#### Childbirth Costs and Childbirth Benefits

In the content analysis, we identified coding criteria for the costs and benefits of childbirth, and the proportions in Tables 2 and 3 refer to the proportion of posts mentioning this category in the total number of posts. It should be noted that the content of posts under research topics varies in length. Some posts involve multiple categories, while others only indicate attitudes without involving any categories. Therefore, the total proportion of categories related to childbirth benefits and costs does not equal 100%.

Among all the categories related to childbirth cost, health risk is the most common category, including severe reactions during pregnancy, physiological pain during childbirth, sequelae of childbirth, and postpartum depression. In addition, limited freedom, energy input, negative influence on the workplace, parenting responsibility, and appearance change are the childbirth costs that people are more concerned about.

Among all the posts, the parenting experience is generally recognized as the greatest childbirth benefit, which includes happiness and love felt in the process of parenting. Compared with childbirth costs, childbirth benefits are mostly subjective feelings and spiritual needs, such as parenting experience, the continuation of life, companionship of children, and the integrity of life. There were only 10/2634 posts (0.38%) that mentioned the objective benefits of bringing up children for the purpose of being looked after in old age.

### Thematic Analysis

#### Overview

Results of thematic analysis generated 4 childbirth concepts from all posts: (1) amplified perception of childbirth risk; (2) hidden childbirth experience and childbirth knowledge spread through informal channels; (3) gender equality and childbirth trap; and (4) the deification of the mother figure. Each theme is presented with detailed descriptions and illustrative examples from the posts.

#### Amplified Perception of Childbirth Risk

In the discussion under the topic, the frequency of mentioning childbirth costs is much higher than that of childbirth benefits, and the explanation of the former makes it easier to attract the attention of the audience and get more likes. Taking the cost of health risks as an example, many users share their personal childbirth experiences, and the real delivery process highlights the physiological pain of childbirth.

I felt regular contractions at two o’clock in the morning when I gave birth to my daughter. When my cervix dilated to about 2cm, I was extremely painful. When it dilated to about 3cm, I couldn’t bear it, so I asked for painless childbirth. After the injection of epidural, I felt tired and breathless without any energy. My head went blank and I couldn’t help to trembling. There was no way but call an anesthesiologist to stop the epidural. Then, I couldn’t feel anything but the pain of contractions. On the second day after I was discharged from the hospital, my lateral incision broke along the suture and became inflamed, so it had to be sutured twice. It was my second time in the delivery room. But this time, I had to have a suture without anesthetic. It was so painful that I even wanted to die and would never forget that feeling in my life.

Poster and Time: XXXXAX, November 1, 2020, 13:42:03

These negative posts may make many unmarried and childless women fear giving birth and then show resistance to childbirth. In addition to the pain and discomfort of pregnancy and childbirth, the complications of pregnancy strengthen the negative perceptions of netizens about childbirth.

Some worries include severe morning sickness during pregnancy, frequent urination at night, a sense of breathlessness as the stomach gets bigger, overwhelming back pain after sitting or standing for a long time in class, too much or too little fetal movement...

Poster and Time: LLL, September 29, 2020, 20:09:01

Pregnancy makes pelvic floor muscles loosen and natural labor will cause bladder prolapse, which takes a long time to heal.

Poster and Time: QSXX, June 24, 2022, 14:02:12

Pregnancy discomfort reactions and recovery processes after childbirth are common experiences for pregnant women, and these symptoms vary in different pregnancy periods and for different individuals. However, many posts emphasize that childbirth will inevitably bring significant health effects and attribute some individuals’ postpartum health changes to childbirth.

Childbirth is a gamble, and it is common to make you get out of shape and have urinary incontinence. Apart from that, you may suffer from some lifelong diseases such as diabetes and eclampsia. Two of my relatives and friends have systemic lupus erythematosus (SLE) after delivery.

Poster and Time: MDAY, October 29, 2020, 00:46:09

Childbirth is a gambling which risks a mother’s life to deliver a baby. Even if it is a successful delivery process, the harm to women is irreversible. My mother used to be a long-distance runner, who is even stronger and healthier than most men. But now this 50-year-old woman still feels cold in winter even when we use many ways to get her warm by opening air conditioner and underfloor heating.

Poster and Time: PER, June 21, 2020, 09:46:47

By browsing so many negative posts listed under this topic, viewers are inclined to think that “childbirth must be painful and will bring negative effects to the body.” In addition to physical health, limited freedom and influence on occupation are important childbirth risks. Without correct guidance, these posts will increase viewers’ perception of childbirth risk to a certain extent and then prevent their willingness from giving birth. Of all the posts, 34/2634 (1.29%) posts mentioned the negative impact of browsing this discussion.

I feel very scared when I view this topic by accident. It seems that I fear marriage and childbirth. I suddenly want to withdraw my couple seeking post. Well, it’s fine to just live alone.

Poster and Time: JDXJ, February 7, 2021, 21:21:59

Whenever I have doubts and shake my faith in marriage and childbirth, I will find this topic to see the advice of my predecessors, and I think I will be safe without marriage and childbirth.

Poster and Time: WAQDZ, April 15, 2022, 18:38:57

#### Hidden Childbirth Experience and Childbirth Knowledge Spread Through Informal Channels

On the one hand, the deviation in viewers’ perception of childbirth risks, especially health risks, comes from the influence of the spread of network information, and on the other hand, it is due to the lack of systematic, complete, and scientific childbirth knowledge. In the posts, many users expressed their lack of relevant knowledge, suggesting that the popularization of childbirth knowledge in schools and society, especially women’s childbirth health, is still insufficient.

There are countless books on the market that teach people how to have a healthy and intelligent child from conception, but few books tell people what damage childbirth will cause to women’s bodies and how to minimize this damage as much as possible. China’s policymakers attach great importance to prenatal and postnatal care. There are all-around and multilevel support means from card establishment during pregnancy, and prenatal care check-ups to breastfeeding and pediatric health care after delivery. But there is almost no means of support for the physical and mental health of mothers. I think this is a result caused by the whole society, paying too much attention to children and neglecting mothers.

Poster and Time: OYSS, July 17, 2020, 11:43:46

Childbirth is an important event that most women in China will experience, and its specific process and health risks rarely appeared in the public field in the past. From the perspective of older females in traditional society, they were ashamed to discuss the childbirth process and its sequelae. Moreover, because every woman in society has to experience this kind of pain, there is no need to emphasize it. Therefore, women are less likely to get relevant experience from relatives and older individuals.

Until the moment I give birth, I never know what it means to my body. Before this, no relatives and friends who become mothers around me have talked about this topic with me. I remembered that in college, I asked my cousin-in-law who gave birth to a child this question and she replied: I feel like I have grown up all of a sudden. When I was a graduate student, I asked my cousin, and she replied: You will know the answer when you deliver a baby—maybe she is shy or maybe she doesn’t know where to start to answer my question.

Poster and Time: PAS, December 5, 2020, 04:14:16

There are various reasons why women lack knowledge about health risks during pregnancy and delivery. For a long time, the whole society has had an insufficient understanding of women’s childbirth, as pointed out by some posts.

For a long time, we have not been allowed to talk about our bodies in public, and some details and experiences including the physiological period, pregnancy and childbirth, are regarded as taboo. But, if we don’t talk about it, we can’t communicate with each other, let alone attract a public concern in society. And if we don’t pay attention to our own body and mind, no one will.

Poster and Time: MMJJ, December 13, 2020, 17:16:08

#### Gender Equality and Childbirth Trap

With the progress of modernization, a growing number of women have gained access to education, actively participated in employment, and started to seek fair labor remuneration and equalized social status. All these factors combined have led to the rise of feminism. Feminism strives to attain gender equality and promote equal rights, opportunities, social recognition, and space for development for all family members, irrespective of gender. The most significant biological difference between men and women lies in the distinct reproductive function of women. Women’s reproductive behavior is a key factor contributing to inequality between men and women, manifesting in various aspects such as access to educational resources, career opportunities, and labor remuneration. Consequently, in the online public opinion environment, discussions related to feminism are often sparked by the topic of childbirth.

Under the research topic, 172/2634 (6.53%) posts mentioned content related to feminism, covering aspects such as unequal reproductive responsibilities between men and women, protection of women’s rights, gender discrimination, abortion rights, and reproductive rights. Among these, 115 posts expressed a negative attitude toward reproduction. In these posts, the reproductive function was no longer perceived as a biological advantage or a gift to women but rather as a negative factor hindering women from pursuing personal development and achieving self-fulfillment. The reason is that the career development of many professional women often comes to a standstill during childbirth, especially during the postpartum period. After giving birth, their incomes suffer as well because a significant portion of their energy has to be invested in caregiving. This has a notable negative impact on those who seek freedom, equality, and socioeconomic status as modern, independent females.

Childbirth has made me a complete feminism and understand the plight of women who can only fight back by not having children. At least at this stage, feminism and childbirth are still at odds with each other. The enormous amount of energy and time required to bear and raise a child is an exponentially increasing workload that has been placed on women for a long time as a ‘punishment for motherhood’. Women are tied to unrecognized labor, and their status is naturally inferior. In addition, there is no security. Now the only option for women to achieve equal economic status is not to have children, not to take on this unrecognized part of the work, but to fight for rightful social status.

Poster and Time: TTZ, October 15, 2021, 17:53:04

In addition to the reality of career stagnation and the unrecognized value of domestic work, the collected posts argued that childbirth can have other negative spiritual effects. These include the identity shift after reproduction, which can confuse women’s self-perception and a lack of self-subjectivity. Moreover, in some cases, childbirth even becomes a tool for society to discipline women.

Marriage and childbirth mean disciplining and reshaping women. The gender concept tends to be traditional, and what once believed, insisted on, and pursued may be annihilated in the trivialities of life and other people’s demands. I fear that kind of change is irresistible, and the fear of losing a part of myself makes me resistant to marriage and childbearing.

Poster and Time: YSBG, July 13, 2020, 00:36:04

Some posts even expressed the belief that childbirth has become a kind of original sin for women, and it does not bring any benefits to them.

Having children is a life choice. In a male-dominated society, childbearing is a disaster for women.

Poster and Time: WT, October 7, 2020, 21:10:08

A pair of bulging breasts and a uterus is the sole source of modern female woe, in a society marinated in misogynistic thinking.

Poster and Time: PLPLB, June 27, 2022, 14:22:05

Admittedly, many of the posts were, to some extent, radical. However, it is essential to consider that the subject of female fertility discourse has been historically obscured for so long that, once people gain the right to free speech, they may resort to language for a thorough emotional release. While women are expressing their repressed feelings, the court of public opinion can influence women who are not married or pregnant, leading to negative evaluations of marriage and fertility.

These feminism-related posts intensively conflict with traditional concepts. In agricultural societies, reproduction and fertility are among the most important values attributed to women in the family. Although modern women are gradually finding new sources of value from education, careers, and society, and to some extent, detaching their self-worth from reproduction, traditional cultural evaluations are slow to change. Many posts also indicated that fertility behavior is subject to social and family pressure.

For a woman who is about to enter her 30 s, the real feeling is that no one (especially the elders) cares about what you want to do in the future, whether you are happy with your life or what your career plan is, but only when you are going to prepare for pregnancy? When are you going to have a baby? It seems that only having a child is the most important thing in your life, and your life is not complete without it!

Poster and Time: BLZ, December 31, 2021, 03:45:34

My mother’s classic quotes are ‘A woman is not complete without a child; bearing no child is a waste of womb; at least have one child, otherwise there is no point in living; a woman will definitely be miserable if she does not have a child......’ In the eyes of traditional women like my mother, the major prerequisite for a woman to have value is that she must have children, otherwise she will not be happy in any way.

Poster and Time: SAN, October 4, 2020, 19:39:56

#### The Deification of the Mother Figure

The image of motherhood is often perceived by society as great, loving, and sacrificial. Becoming a mother is also a process of self-growth for many women. Self-growth is one of the most frequently cited childbirth benefits under the topic, and it includes the maturation of character, usually embodied in the shift from self-centeredness to child-centeredness. However, this shift can sometimes be overdone and give rise to a lack of self.

Childbearing means that henceforth she will literally become a so-called attachment to the family in the eyes of the world; it means that henceforth she is better off realizing her value as a virtuous wife and mother than as an independent human being in the eyes of the world.

Poster and Time: HEL, November 21, 2020, 23:23:08

In a life of constant reinforcement, people take the phenomenon that a mother gives 100% of her love and care her children for granted. However, the truth is that the independence of the woman herself has been overlooked. Imagine you are going to visit a friend who recently gave birth to a baby, will you prepare a gift for the baby?

Poster and Time: DHAXX, November 22, 2020, 10:39:39

It is not difficult to see that this neglect of the mother’s subjectivity is socially structured, with all the attention from the mother herself to family relatives and even society revolving around the child. The lack of maternal subjectivity is based on the cultural notion that it is obligatory and common for mothers to sacrifice for their children. When a family accommodates a child, the child becomes the most important being in the family. When a woman becomes a mother, society expects her to become a great mother. Being shackled by such an expectation, the woman’s sacrifices become deserved and her feelings become secondary.

A woman who is married and has children is taken away from the immunity of making mistakes and is by default an indestructible file. She is supposed to understand everything and bear all the hardships. Those who have experienced it know that it is anguished, but she is not allowed to cry out in pain.

Poster and Time: LHQ, February 21, 2021, 20:17:02

The deification of the mother figure is also reflected in society’s belief that women should do their best in all aspects of the childbirth process for the good of their children and that it is common to sacrifice a mother’s time, energy, and even health to do so.

I think society has a very strong tendency towards perfectionism in the mother-infant relationship. Anytime you see contents like mom’s hands make the best supplements (what about dad’s or grandparents’ hands?) Even if it’s just bottle-feeding, it’s believed that mom ought to do it herself (does it truly have that much of a negative impact if someone else does it?) As a woman, I think we need to realize that these requirements may not actually be very scientific. Most people grew up with a handful of 100 in examinations, so how is possible that when you get to raise a child you suddenly become perfect at everything?

Poster and Time: AHJM, August 15, 2021, 19:32:23

The high expectations of society for mothers may be internalized and transformed into high standards for themselves. Admittedly, it has a positive impact from this aspect, as mothers would actively seek to grow if they want to take better care of their children or use themselves as role models.

I myself do not truly like little kids, but after becoming a mother, I always want to set an example so that my baby feels that his mother is also an awesome person.

Poster and Time: DAR, June 23, 2020, 22:17:02

After you lost your temper and lashed out at your child, you are likely to think you are not a competent mom after calming down, and cringe at not being able to control yourself. Then, you will naturally want to be better in order to be a role model for your child. As a mother of a three-and-a-half-year-old kid, childbirth for me was something I needed to become stronger, better, and more mature.

Poster and Time: Sayly, August 1, 2020, 14:17:49

While such high standards can promote women’s self-growth to some extent, these standards may bring about negative emotions, such as anxiety, if they are too high to meet. For example, some posters indicated that they would feel guilty for not loving their children enough or for not prioritizing them above all else.

I was recently reading The House on the Slope, and I clearly remembered that the main character, Risako, faced the challenge of not having enough breast milk after becoming a new mother. The idea of exclusive breastfeeding instilled in her by people around her made her feel less confident, doubt, and deny herself. There was even one time she became tired of parenting and only wanted to escape. Is it true that if you cannot breastfeed exclusively, you are not a good mother and therefore not worthy of being a human being?

Poster and Time: XRK, September 29, 2021, 12:28:33

I don’t want to be a mother anymore. It’s too hard. I always unintentionally hurt my child, such as underdressing her, overcovering her, holding her incorrectly, accidentally bumping her, and not being able to give her a comfortable living environment. She cries, and in many cases, I don't even know why.

Poster and Time: TJDSXJ, December 8, 2020, 21:03:02

The unreasonably high standards dissuade women who are not married or pregnant. Many of the users posting are not afraid of bearing children but are apprehensive about not being able to be a “perfect” mother and not being able to take responsibility for their children’s upbringing and education.

I don’t have confidence that I can provide a good enough life for him, that I have enough patience to educate him, and that I can give up something for him without regret.

Poster and Time: YKDGJ, May 5, 2021, 23:56:59

## Discussion

### Principal Findings

Based on the representative topic of childbirth, this study analyzed the ecology of online public opinion on childbirth using a mixed method of content and thematic analyses. It has been found that women’s attitudes toward childbirth were generally neutral (1060/2634, 40.24%) or negative (1051/2634, 39.90%), with only a few showing a positive stance (523/2634, 19.86%). Messages with negative attitudes received more follows and likes. This finding is consistent with existing Chinese literature analyzing childbirth willingness from online texts [25,26]. Previous studies showed that there are often more negative posts on fertility-related topics on Weibo [14], TikTok [27], WeChat [28], and other social media platforms in China. By analyzing the trend of women’s attitudes toward childbirth, we found that the proportion of posts expressing a negative attitude fluctuates and rises over time. According to previous reports [26,29,30] and the analysis of this study, we believe that both environmental factors and personal subjective factors interact to affect women’s attitudes toward childbirth. On the one hand, environmental factors, including rising housing and living costs, intense competition in child education, and the job market, as well as increased work pressure, lead young people to choose to have fewer children. On the other hand, from the perspective of subjective factors, the need for self-realization has motivated women to pursue higher education and success in their careers, leading to a delay in the age of marriage and childbirth, and a choice to have fewer children [31].

Childbirth costs and benefits are common concerns for women. In this study, health risk, restricted freedom, and energy input were the most frequently mentioned aspects in the broad topic of childbirth costs. This finding differs from Gao [25], who used textual analysis on childbirth-related content crawled from other social media. In our study, health risks were discussed more frequently and received more attention, whereas Gao [25] found that economic impacts such as financial investment were more important. The difference may be attributed to the fact that different social media have different audiences and public opinion climates. Compared with other social media such as Weibo and WeChat, Douban’s users have a higher proportion of female users. Currently, Chinese families generally have more financial responsibilities borne by men, while most of the users posting under the topic of childbirth are women. Therefore, the collected posts were mostly generated from women’s perspectives, which naturally include health risks, restricted freedom, and energy input. Turning our attention to childbirth benefits, we found that posters focused on spiritual needs and subjective feelings, such as parenting experience, self-growth, and the continuation of life. However, only 10/2634 posts (0.38%) mentioned the objective benefits of bringing up children for the purpose of being looked after in old age. It shows that the childbirth concept of urban women has gradually changed from the traditional ones of “passing on the family line” and “bringing up children for the purpose of being looked after in old age” to the modern ones of “emotional experience,” “spiritual needs,” and “the pledge of love.” This finding is consistent with the findings of previous studies investigating childbirth motivation and childbirth willingness [4].

### Concept of Childbirth

Childbirth was described as a painful process in social media. Some women have pregnancy complications. Physical pain during childbirth is a major source of fertility fear for young women [32]. Some social media users vividly describe their childbirth experience in detail. It makes viewers feel like they went through the same situation personally. Negative events could be widely spread on the internet and attract public attention [33]. This negativity can affect social media users through emotional contagion [34]. In line with previous findings on the relationship between social media and risk perceptions [28], this paper concludes that social media indeed increase women’s childbirth risk perceptions. Taking a step further, this paper also provides specific examples for the expansion of women’s risk perception.

This study also finds that many women lack comprehensive and scientific knowledge about the health risks of pregnancy and delivery. Without systematic and scientific health knowledge, many women are not only unable to properly cope with potential health risks but also have a misleading perception or wrong expectations of childbirth risks, thereby increasing their anxiety about childbirth due to the information received through informal channels. At present, the internet has become an essential means for women to gather knowledge about childbirth health [35,36]. However, most of them did not discuss the information they retrieved from the internet with their health care professionals [37,38]. Social media such as Twitter, Reddit, and Facebook provide forums for private citizens to freely express their views, including those about medicine and health care. Yet, the content disseminated through websites and online communities is largely unregulated [39]. This situation highlights that the internet, as an informal communication channel, may unfavorably bias women’s fertility perceptions. Currently, network information has become a crucial means for people to acquire knowledge about childbirth, with some blogs focused on the popularization of childbirth knowledge and sharing childbirth experiences gaining widespread attention on the internet. Under this topic, among the top 20 posts, 3 (15%) are about personal health changes and sharing information about breast milk. This reflects the prevalence of personal blogs discussing childbirth health knowledge and also underscores the demand for this type of information among the audience.

The discussions on the topic of childbirth illustrate that modern women are influenced by both traditional and modern fertility concepts. On the one hand, women seeking independence and equality are easily swayed by internet trends, believing that childbirth contributes to gender inequality and impedes personal self-fulfillment, leading to resistance to marriage and childbirth. On the other hand, they face significant pressure from traditional family values, as mentioned earlier. The collected posts reflect that the value of childbirth to women has become ambiguous and contradictory as the approaches to women’s self-worth have broadened. Zhang et al [27] also pointed out the ambivalent mindset of the new generation of women regarding childbirth and self-evaluation. This shift in gender concepts often has a negative impact on fertility [40].

In addition, the shift in women’s family identity brought about by childbirth leads many of them to feel a lack of subjectivity. The image of motherhood is often perceived by society as great, loving, and sacrificial. Most women believe that motherhood is a rite of passage for women, characterized by the transition from a selfish child to a selfless adult [41]. The maternal norms constructed by society embody characteristics of self-sacrifice. All mothers are expected to adhere to the moral standards of being a “good” mother. These norms make some mothers feel uncomfortable and distressed [42,43]. This kind of sacrifice has also faced criticism from some feminists because sacrifices made for children and partners might perpetuate oppressive gender norms, burdening women and further relinquishing their freedom [44,45].

This article reveals that the elevated social expectations imposed on mothers can be internalized, leading to the establishment of high standards for themselves. While this motivation drives them to pursue self-growth, it simultaneously triggers anxiety about parenting. These heightened social expectations have the potential to not only induce anxiety in mothers who have experienced childbirth but also instill fear among those who have not given birth.

### Strengths and Limitations

To our knowledge, only a limited number of previous studies have explored the influence of social media on women’s perceptions of childbirth. Among them, even fewer have delved into the analysis of user-generated text on social media platforms. Instead, some studies opted to assess the sentiments of their study participants through questionnaires and interviews. In this study, we conducted a thorough analysis of the posts and discussions shared by users on Douban, a social media platform known for attracting a large population of highly educated young individuals. This user base contributes to the clarity, completeness, and rationality of the points expressed in the discussions. The chosen research method enables us to gain new insights, particularly highlighting how the deification of the mother figure may result in elevated public expectations, pressuring women to sacrifice themselves to attain the ideal of a perfect mother. Women may be hesitant to become mothers as they fear losing their sense of self. Another noteworthy insight is that longstanding social and cultural biases in China may have hindered open discussions on the childbirth process and its health implications, consequently contributing to a lack of comprehensive childbirth knowledge among young women.

The findings of this study should be considered in light of certain limitations. The majority of Douban users reside in urban areas, limiting the generalizability of the results to rural women who may have different perspectives on childbirth. Future research on the attitudes and concepts of childbirth among rural women is warranted. However, previous studies have indicated that the internet’s development also influences the gender role concepts of rural residents, transitioning from traditional to modern, with women being significantly more affected than men [9]. The suggestion is made that the influence of cultural concepts via the internet is present in both urban and rural areas, and analyzing urban women’s concepts is valuable for understanding those of rural women. Additionally, the gender of all users in this study could not be determined. However, based on the content of the topic and the detailed study of part of the selected sample, it is considered that the majority of the sample consisted of females. While it cannot be ruled out that a few users might be male, it is considered that this would not significantly alter the analysis results and findings.

### Conclusion and Suggestions

This study discovered that users generally held neutral or negative attitudes toward childbirth, with fewer expressing a positive stance. Additionally, posts with a negative attitude garnered more attention and likes. Moreover, there was an observed increase in posts with negative attitudes in recent years compared with earlier years. A significant number of young women lack comprehensive and scientific knowledge about the health risks associated with pregnancy and delivery. They tend to rely on the internet to gather relevant information. However, the internet, functioning as an informal communication channel, may inadvertently skew women’s perceptions of fertility. As women’s socioeconomic status has elevated, there is a redefinition of the value attached to childbirth. In Chinese society, women often encounter greater restrictions on their freedom and are required to invest more energy in childcare compared with men, leading them to hesitate or even resist childbirth [46,47]. Moreover, the structural neglect of mothers’ needs and desires in Chinese society creates a perception among women that they might lose themselves due to childbirth and child-rearing, contributing to their hesitation or resistance to giving birth.

The study findings highlight the importance of monitoring public expressions on the internet, offering guidance to women seeking information on pregnancy and delivery, and assisting them in developing a scientific understanding of childbirth. Furthermore, enhancing the public childcare system, safeguarding women’s rights, and creating a supportive societal environment for mothers could potentially contribute to an increase in women’s fertility desires.
